# Nitric Oxide Manipulation: A Therapeutic Target for Peripheral Arterial Disease?

**DOI:** 10.1155/2012/656247

**Published:** 2012-03-20

**Authors:** Gareth Williams, Xu Shi-Wen, David Abraham, Sadasivam Selvakumar, Daryll M. Baker, Janice C. S. Tsui

**Affiliations:** ^1^Division of Surgery & Interventional Science, University College London, Royal Free Campus, London NW3 2QG, UK; ^2^Centre for Rheumatology & Connective Tissue Disease, University College London, Royal Free Campus, London NW3 2QG, UK; ^3^Department Of Surgery, Lister Hospital, Coreys Mill Lane, Stevenage, Hertfordshire SG1 4AB, UK

## Abstract

Peripheral Arterial Disease (PAD) is a cause of significant morbidity and mortality in the Western world. Risk factor modification and endovascular and surgical revascularisation are the main treatment options at present. However, a significant number of patients still require major amputation. There is evidence that nitric oxide (NO) and its endogenous inhibitor asymmetric dimethylarginine (ADMA) play significant roles in the pathophysiology of PAD. This paper reviews experimental work implicating the ADMA-DDAH-NO pathway in PAD, focussing on both the vascular dysfunction and effects within the ischaemic muscle, and examines the potential of manipulating this pathway as a novel adjunct therapy in PAD.

## 1. Introduction

Peripheral arterial disease (PAD) is a disorder characterised by the progressive occlusion of the large- and medium-sized arteries outside of the cardiopulmonary and cerebral vascular systems. It causes considerable morbidity and mortality, affecting over 2.7 million individuals in the Western world [1] and most commonly manifests itself in the reduction of blood flow to the lower limbs, which in severe cases lead to chronic pain, gangrene, and eventually limb loss. Risk factors include tobacco smoke, diabetes mellitus, hypertension, and hypercholesterolaemia.

At a molecular level, dysfunction of the vascular endothelium, in particular, disruption of the nitric oxide (NO) pathway [2], plays a significant role. NO is a vasodilator synthesized by the enzyme nitric oxide synthase (NOS) [3] and has a number of important endocrine and paracrine effects, including reduction of vascular smooth muscle tone; inhibition of platelet adhesion and aggregation; suppression of inflammatory mediators; inhibition of smooth muscle proliferation and migration; and promotion of endothelial survival and repair [4].

There have been significant efforts to elucidate the pathophysiological role of the NO pathway in cardiovascular diseases, with recent discoveries of endogenous NO inhibitors adding further complexity [5], but this has yet to be translated into clinically significant therapies.

This paper aims to outline the current knowledge on NO pathway dysfunction in PAD, including recent developments in understanding the role of asymmetric dimethylarginine (ADMA) and dimethylarginine dimethylaminohydrolase (DDAH); it will also explore potential therapeutic strategies based on the manipulation of this pathway.

## 2. The Nitric Oxide Pathway

NO is synthesised from L-arginine by NOS, of which there are three isoforms [6]: NOS I or neuronal NOS (nNOS) was originally isolated from rat and porcine cerebellum; NOS II or inducible NOS (iNOS) from activated macrophages; NOS III or endothelial NOS (eNOS) from endothelial cells.

**Figure 1:**
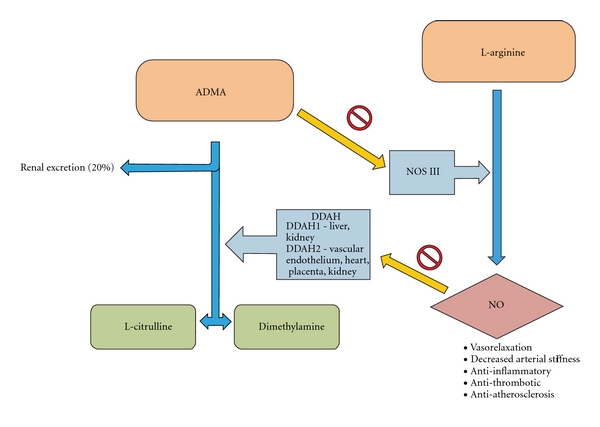
The nitric oxide pathway.

All 3 NOS isoforms are competitively inhibited by the methylarginines ADMA and monomethyl-arginine (L-NMMA) which are by-products of protein degradation [7]. Both ADMA and L-NMMA are predominantly broken down by the enzyme DDAH into L-citrulline and dimethylamine [8], of which there are two isoforms [9]: DDAH I, found predominantly in tissues expressing NOS I, liver, kidney, and lung; and DDAH II, found in tissues expressing NOS III and NOS II, vascular endothelium, heart, placenta, and kidney. Both isoforms have been found to be expressed in vascular endothelium [9] (Figure 1).

ADMA's role in vascular endothelial dysfunction was first described by Vallance et al. in patients with end-stage chronic renal failure, whose serum ADMA is raised due, in part, to decreased renal clearance [10]. Further studies in similar patients have shown a positive correlation between ADMA levels and cardiovascular morbidity and mortality [11]; whilst reduction of ADMA levels by renal dialysis helps to restore endothelial function [12].

Other studies have demonstrated a correlation between ADMA accumulation and cardiovascular risk as well as other disease states [5]. Lu et al. showed that serum ADMA levels have a positive correlation with the severity and extent of coronary artery atherosclerosis [13]; Worthmann et al. demonstrated a similar correlation between ADMA levels and adverse prognosis in ischaemic stroke [14]; and ADMA has been found to have significant roles in renal disease [15], pulmonary artery hypertension [16], and erectile dysfunction [17].

The accumulation of ADMA and consequent endothelial dysfunction, increased systemic vascular resistance, and increased systemic blood pressure have been shown to be due to reduced DDAH expression [18]. Upregulation of DDAH I and DDAH II in human umbilical vein endothelial cells (HUVECs) by adenoviral vectors leads to a fall in ADMA and an increase in NO [7]. Similarly, upregulation of DDAH I and II in DDAH I^+/−^ mice was found to attenuate the cardiovascular stress response, enhance acetylcholine-mediated relaxation, and counteract the response to excess ADMA; DDAH upregulation helps to reverse the cardiovascular dysfunction inherented in these animals [7].

However, DDAH I appears to be the main isoform responsible for ADMA metabolism: DDAH I knockout mice demonstrated significantly raised ADMA levels and systolic blood pressure compared to wild types, despite DDAH II expression remaining normal [19].

These studies suggest that DDAH manipulation may be a useful tool in the treatment of cardiovascular disease.

## 3. Peripheral Arterial Disease (PAD)

In PAD, lower limb ischaemia results in intermittent claudication: this describes pain in the affected muscle groups brought on by exercise and relieved by resting. In more severe PAD, critical limb ischaemia (CLI) occurs: the patient experiences chronic pain at rest, with blood flow to the limb compromised to the extent that the affected limb is at risk, with development of ulcers and gangrene.

The Fontaine classification [20] stratifies these symptoms to correlate with severity of the disease (Table 1).

Current therapies for CLI centre on restoring blood flow to the affected limb by surgical and/or endovascular procedures. Unfortunately, of all patients presenting with CLI, only 50% will have disease amenable to revascularisation with 25% requiring a primary amputation. The overall prognosis of these patients is also poor: within a year of presentation, a quarter of the patients would have died, 30% would have had a major amputation, and 20% continue to suffer from CLI [1].

There is evidence that the NO-ADMA-DDAH pathway plays an important role in PAD. Evidence of systemically impaired NO synthesis has been described by Böger et al. who showed urinary nitrate and cGMP excretion to diminish in PAD patients as the disease progressed [2]. This group also demonstrated that intravenous L-arginine administration induced NO-mediated vasodilation, increasing femoral blood flow in patients with CLI, resulting in improved walking distances and symptom scores [21]. Furthermore, raised plasma ADMA levels have also been shown to correlate with disease severity in PAD patients [5], implicating the involvement of ADMA.

NO also promotes angiogenesis, activating vascular endothelial growth factor and fibroblast growth factor [22]. Administration of NO antagonists to canine coronary ischaemia models has demonstrated that NO is an important mediator of new vessel growth [23]. This mechanism of stimulation of new blood vessel development to supply ischaemic tissue has been proposed as a possible treatment for PAD, but clinical results have so far been disappointing. The formation and persistence of new blood vessels is likely to be multifactorial, involving a complex interaction between chemokines and mechanical forces [24]. In addition, continued exposure to cardiovascular risk factors, such as hypercholesterolaemia and hyperglycaemia, has been shown to have a deleterious effect on the NO pathway [25, 26].

However, the NO-ADMA-DDAH pathway role in PAD is not confined to vascular dysfunction: NO-ADMA-DDAH interaction is known to play significant parts in the function, homeostasis, and repair of skeletal muscle. Weight for weight, this is the most abundant tissue in the lower limb and the majority of PAD symptoms are attributable to skeletal muscle ischaemia; it can almost be regarded as a “target organ” of PAD. Hence, the NO-ADMA-DDAH pathway within ischaemic skeletal muscle represents a significant facet of potential CLI therapy.

## 4. NO in Skeletal Muscle

Skeletal muscle has been shown to express both NOS I and NOS III: NOS I is localized to the sarcolemma, and NOS III associated with mitochondria and microvessels within the tissue [27]. Within skeletal muscle, NO acts as an endogenous modulator of vascular tone [28, 29], neuromuscular transmission [30], muscle contraction [31, 32], muscle metabolism [27] and myogenesis [33]. Its role in the regulation of muscle contraction is complex and is influenced by the muscle fibre type and contraction pattern and frequency involved [31]. NO has a number of effects on skeletal muscle metabolism [27]. It affects glucose metabolism, upregulating glucose uptake and inhibiting glycolysis [34]; it has a direct inhibitory effect on mitochondrial respiration [35]; and it antagonises creatinine kinase activity, decreasing phosphocreatine breakdown, limiting muscle function [36].

NO plays a role in myogenesis as well as repair and regeneration following injury. It has been shown to modulate the inflammatory response to injured skeletal muscle, controlling blood flow to the injured tissue, and affecting subsequent remodelling and repair [37]. In experimental models of muscle crush injury, upregulation of local NO has been demonstrated [38], inhibiting adhesion and inducing apoptosis in inflammatory cells [39]. NOS is activated in response to stretch in skeletal muscle, mediating the release of hepatocyte growth factor and activating mononuclear progenitor cells called satellite cells (SC), which are responsible for post-injury remodelling [40].

Tidball et al. showed that the addition of exogenous NO upregulated the expression of structural proteins within mouse myotubes and found that NOS antagonists inhibited this, suggesting a regulatory role for NO in myogenesis [33].

In addition to local effects, NO appears to play a part in the systemic inflammatory response: experimental rats with peritonitis or septic shock have been shown to have increased expression of NOS in their skeletal muscle [41].

However, NO has also been shown to have deleterious effects, reacting with superoxide anions to form harmful nitrogenous intermediates which are directly toxic to skeletal muscle and inhibit mitochondrial function [41, 42].

## 5. NO in Ischaemia

Impaired NO-mediated vasodilation has been found in postischaemic tissue such as myocardium [43], brain [44], and skeletal muscle [45]. There is a complex interaction between ischaemia and the NOS isoforms, with both up- and downregulation reported in ischaemic models; for instance, rat brain oxygen-glucose-deprivation models have shown NOS II to be upregulated in response to ischaemia, which in turn causes a downregulation of NOS I [46]; however, ischaemic preconditioning of rat liver has been shown to upregulate NOS III [47].

**Figure 2:**
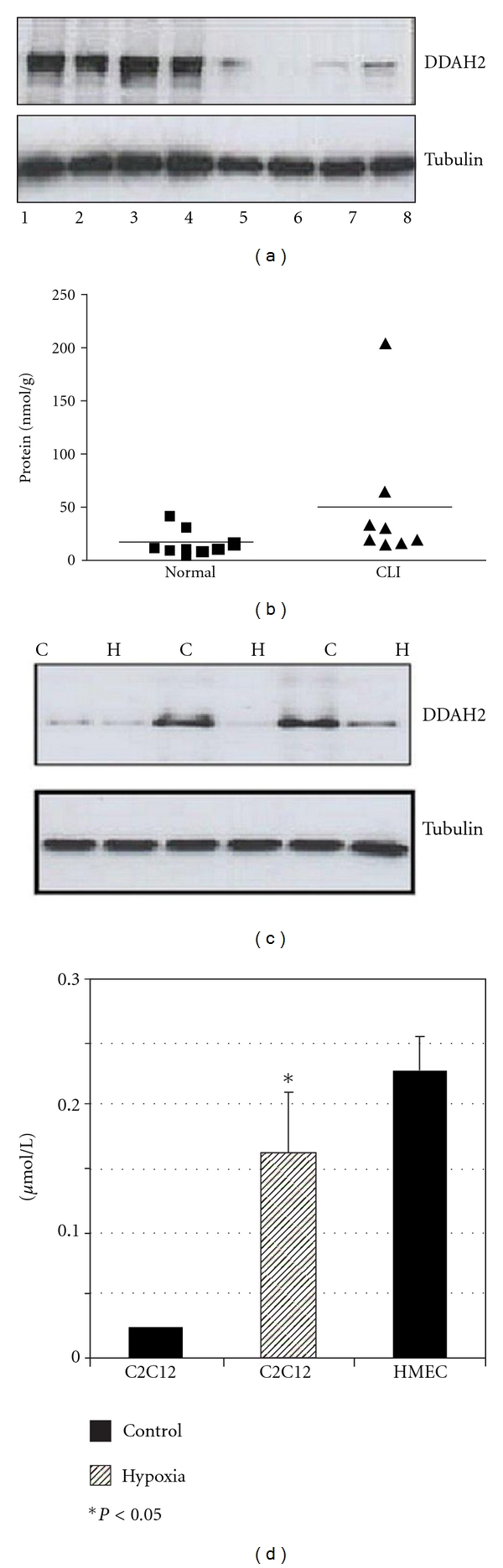
ADMA/DDAH pathway in ischaemic muscle—preliminary data. (a) Western blot showing reduced DDAH2 expression in muscle biopsies from patients with CLI. Lanes 1–4: control, 5–8: CLI muscle. (b) ELISA showing significantly higher ADMA levels in muscle from patients with CLI (*P* = 0.03, Mann Whitney* U* test). (c) Western blot showing reduced DDAH2 expression in hypoxic myotubes (C: control, H: hypoxia). (d) Conditioned medium from hypoxic C2C12 myotubes contained elevated levels of ADMA compared to medium from myotubes cultured in normoxia. (*P* < 0.05, Student′s *t*-test). Medium from HMEC-1 cells was used as positive control.

In skeletal muscle from patients with CLI, we have previously found raised NOS III expression associated with both microvessels within ischaemic muscle sections as well as the muscle fibres themselves [48]. However, this was not associated with an increase in NOS activity. The involvement of endogenous inhibitors may explain this and recent preliminary data suggesting that ADMA levels in muscle homogenates from patients with CLI are raised (Figures 2(a) and 2(b)). In addition, DDAH2 protein levels are also upregulated in these ischaemic muscle biopsies.

To study the potential role of the ADMA/DDAH pathway in skeletal muscle further, we evaluated their levels in myotubes cultured in ischaemic conditions *in vitro*. We found reduced DDAH2 expression in ischaemic myotubes, whilst ADMA levels were elevated in ischaemic myotube conditioned media (Figures 2(c) and 2(d)).

Whilst the majority of studies have focussed on ADMA associated with the vasculature/endothelial cells, our findings from our *in vitro* studies suggest that ischaemic myotubes may be a source of ADMA which can then act in a paracrine fashion on surrounding endothelial cells or in an autocrine manner on myotubes themselves with potentially detrimental effects.

Fiedler et al. found that ADMA impairs vascular endothelial growth factor (VEGF) mediated angiogenesis, and that this was prevented by upregulation of DDAH [49]. Increased levels of ADMA produced by ischaemic muscle may prevent angiogenesis in PAD patients and may be a factor in the failure of angiogenesis induction as a clinical therapeutic approach.

## 6. Manipulation of the ADMA-DDAH Pathway

Control of NO levels by manipulating the ADMA-DDAH-NO pathway represents a significant avenue for potential early PAD treatment.

As L-arginine is the main precursor for NO production (Figure 1), an increase in the availability of substrate ought to result in an increase in NO. However, there is conflicting evidence as to whether L-arginine supplementation has a clinically significant benefit in cardiovascular disease: Sydow and Münzel [50] showed that L-arginine improved radial artery dilatation, in contrast to Walker et al's findings that vasodilatation was independent of oral L-arginine supplementation [51]. In PAD, the Nitric Oxide in Peripheral Arterial Insufficiency (NO-PAIN) study randomized 133 patients with intermittent claudication to oral L-arginine versus placebo for 6 months. Whilst L-arginine supplementation increased plasma L-arginine levels, there was no improvement in NO availability or vascular function. This was in contrast to earlier, smaller studies of shorter duration demonstrating a beneficial effect of L-arginine in PAD patients [21]. A mechanism of “arginine-tolerance” was proposed, in a similar manner to “nitrate-tolerance”, observed in prolonged nitroglycerine use. More disappointingly, whilst both groups in the NO-PAIN study showed improvement in absolute walking distance, the improvement in the L-arginine group was significantly less than the placebo group [52].

An alternative strategy to increase NO levels may be to reduce ADMA levels by enhancing DDAH activity or expression. Nebivolol [53], pioglitazone [54], pravastatin [55], and fenofibrate [56] have all been shown to decrease ADMA by upregulating DDAH *in vitro*. Clinically, Sen et al. demonstrated an increase in circulating NO and a decrease in ADMA, coupled with improvement in symptoms in patients with ischaemic heart disease treated with the selective beta1-adrenergic receptor antagonist nebivolol [47]. Nishiyama et al. found that the statin, pravastatin reduced serum ADMA in patients with cerebrovascular disease [57].

In PAD, upregulation of DDAH at a local level to reduce ADMA levels within the ischaemic muscle may be of benefit in a number of ways: it may improve microvascular function and local tissue perfusion; it may improve the results of therapeutic angiogenesis by reducing the antiangiogenic environment; or it may improve muscle contractile and metabolic function and perhaps reduce tissue damage. In patients with CLI, such strategies may be used as an adjunct to improve the results of revascularisation techniques as well as reduce tissue damage in patients with non-reconstructable disease. They may also be of benefit to patients with earlier disease to prevent disease progression.

## 7. Conclusion

There is considerable evidence that disruption of the NO pathway plays a major role in the pathogenesis of PAD. ADMA in particular, through its actions on NOS III, has been shown to be an important modulator of NO. Early work suggests that local or systemic manipulation of ADMA, by upregulating DDAH, may prove to be a novel treatment for PAD.

## Figures and Tables

**Table 1:** Fontaine Classification: stages III and IV are also known as CLI.

Stage I	Asymptomatic
Stage II	Intermittent claudication, no rest pain
IIa	When walking a distance of greater than 200 m
IIb	When walking a distance of less than 200 m
Stage III	Nocturnal pain and/or pain at rest
Stage IV	Tissue loss: ischaemic ulcers and/or gangrene
